# Intrauterine exposure to di(2-ethylhexyl) phthalate (DEHP) disrupts the function of the hypothalamus-pituitary-thyroid axis of the F1 rats during adult life

**DOI:** 10.3389/fendo.2022.995491

**Published:** 2023-01-13

**Authors:** Érica Kássia Sousa-Vidal, Guilherme Henrique, Renata Elen Costa da Silva, Caroline Serrano-Nascimento

**Affiliations:** ^1^ Faculdade Israelita de Ciências da Saúde Albert Einstein, Hospital Israelita Albert Einstein, São Paulo, Brazil; ^2^ Laboratório de Endocrinologia Molecular e Translacional (LEMT), Escola Paulista de Medicina (EPM), Universidade Federal de São Paulo (UNIFESP), São Paulo, Brazil; ^3^ Instituto de Ciências Ambientais, Químicas e Farmacêuticas (ICAQF), Departamento de Ciências Biológicas, Universidade Federal de São Paulo (UNIFESP), Diadema, Brazil

**Keywords:** phthlates, DEHP, endocrine disruptors, hypothalamus-pituitary-thyroid axis, intrauterine period, DOHaD

## Abstract

**Introduction:**

DEHP is an endocrine disruptor widely used in the production of malleable plastics. DEHP exposure was associated with altered hypothalamic-pituitary-thyroid (HPT) axis function. Although previous studies reported deleterious effects of DEHP exposure during the intrauterine period, few studies have evaluated the direct effects triggered by this endocrine disruptor on the offspring animals' thyroid function. This study aimed to investigate the impact of intrauterine exposure to DEHP on the HPT axis function programming of the offspring animals during adulthood.

**Methods:**

Pregnant Wistar rats were orally treated with corn oil or corn oil supplemented with DEHP (0.48 or 4.8 mg/kg/day) throughout the gestational period. The offspring rats were euthanized on the 90th postnatal day. Hypothalamus, pituitary, thyroid, and liver were collected to analyze gene expression and protein content through qPCR and Western Blot. Blood was collected to determine TSH and thyroid hormone levels through fluorometric or chemiluminescence immunoassays.

**Results:**

In the adult F1 female rats, the highest dose of DEHP decreased TSH serum levels. In the thyroid, DEHP reduced the gene expression and/or protein content of NIS, TSHR, TG, TPO, MCT8, NKX2.1, PAX8, and FOXE1. These data are consistent with the reduction in T4 serum levels of the F1 DEHP-exposed female rats. In the liver, DEHP exposure increased the mRNA expression of *Dio1* and *Ttr*, while the highest dose of DEHP reduced the mRNA expression of *Ugt1a1* and *Ugt1a6*. Conversely, in the F1 male adult rats, TSHB expression and TSH serum levels were increased in DEHP-exposed animals. In the thyroid, except for the reduced protein content of TSHR, none of the evaluated genes/proteins were altered by DEHP. TH serum levels were not changed in the DEHP-exposed F1 male rats compared to the control group. Additionally, there were no significant alterations in the expression of hepatic enzymes in these animals.

**Discussion/Conclusions:**

Our results demonstrated, for the first time, that intrauterine exposure to DEHP disrupts the HPT axis function in male and female offspring rats and strongly suggest that DEHP exposure increases the susceptibility of the offspring animals to develop thyroid dysfunctions during adulthood.

## 1 Introduction

Phthalates are plasticizers used in producing polyvinyl chloride plastics and several manufactured goods, such as toys, food packaging, and medical devices. Phthalates are not covalently bound as plasticizes, and thus can easily leach into the water, food, and environment (1, 2).

Di(2-ethylhexyl) phthalate (DEHP) is the most common member of the class of phthalates, and several studies indicated that humans are highly exposed to DEHP (3, 4). Indeed, DEHP exposure was considered a potential risk to human health (5–7). The estimated daily intake of DEHP for adult humans was 0.5-30 μg/kg/day, although some studies have reported higher levels of exposure to this phthalate (8–10). Once in the body, DEHP is metabolized to mono(2-ethylhexyl) phthalate (MEHP), a highly toxic compound (11).

DEHP and MEHP are compounds that interfere with the endocrine function in humans and other animals (12). It is worth noting that the thyroid hormones control metabolic processes essential for normal development and growth (13–15). Moreover, the thyroid hormones are transferred through the placenta to the fetal compartment during the intrauterine period, and the thyroid hormones’ actions are crucial to the development of the central nervous system (16–18).

Although it has been suggested in several epidemiological studies, the negative association between thyroid hormone serum levels and DEHP exposure is still controversial in humans (19–22). On the contrary, the disruptive effect of DEHP exposure on the thyroid function of the most susceptible individuals, such as newborns, children, and pregnant women, was extensively reported in the literature (23–25).

In animal models, the direct and chronic exposure to high doses of DEHP (250 to 750 mg/kg/day) was related to increased serum levels of TRH and TSH, decreased thyroid hormones serum concentration, altered expression of genes/proteins involved in the thyroid hormone synthesis, altered peripheral metabolism and impaired plasma transport of thyroid hormones to the target tissues (26–28).

It is well known that the intrauterine period is essential for programming health or disease during adulthood (29–31). Nevertheless, although it has been described that *in-utero* exposure to DEHP was related to neurobehavioral and neurodevelopment impairments due to alterations in thyroid homeostasis (32, 33), limited studies investigated the effects of DEHP early-life exposure directly in the thyroid function of the offspring animals. Therefore, this study aimed to evaluate the impact of intrauterine exposure to DEHP in the programming of the HPT axis of the adult F1 rat offspring.

## 2 Material and methods

### 2.1 Animals and experimental protocol

**Figure 1:**
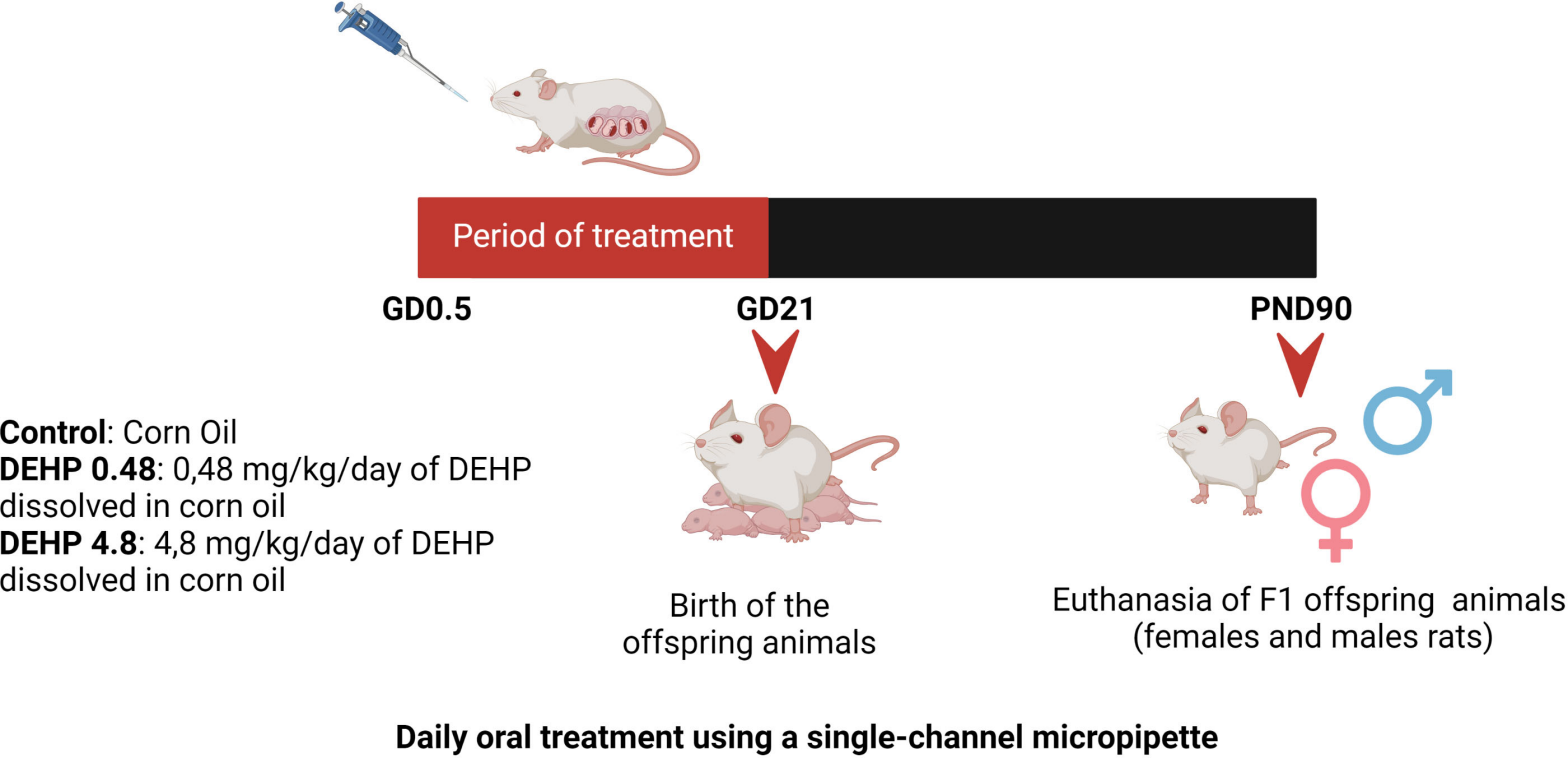
Schematic representation of the experimental protocol. Pregnant Wistar rats were randomly divided into 3 experimental groups: Control, DEHP 0.48 mg/kg/day and DEHP 4.8 mg/kg/day. Pregnant rats orally received corn oil (control) or corn oil supplemented with DEHP (0.48 or 4.8 mg/kg/day) throughout pregnancy (GD0.5 – GD21). After birth, F1 offspring male and female rats were maintained with normal rat chow and filtered water. The offspring animals were anesthetized and euthanized in the PND90. Created with BioRender.com.

Virgin female and male Wistar rats were obtained from the Animal Breeding Centre at the Institute of Biomedical Sciences, University of Sao Paulo. The animals were maintained in polysulfone rat cages in the Experimental and Training Center of the Hospital Israelita Albert Einstein at constant temperature (23 ± 1°C), 12:12-h light-dark cycle schedule, and fed with rat chow (NUVLAB, CR1; Nuvital, Brazil) and water (polysulfone bottles) *ad libitum*. The female and male rats were mated, and the presence of spermatozoa in the vaginal smear was defined as the first day of gestation. Thereafter, each pregnant rat was randomly assigned to one of 3 treatment groups: control, 0.48 mg/kg/day (DEHP 0.48), or 4.8 mg/kg/day (DEHP 4.8). Four pregnant rats were used in each experimental group. Each pregnant rat had one litter of 10-12 animals per group. The number of offspring animals did not vary among the groups. DEHP was dissolved in corn oil (**Figure 1**). The treatment doses were based on the NOAEL dose of DEHP, which was defined as 4.8 mg/kg/day (34, 35).

From the first day of gestation until the birth of the pups, pregnant dams were orally dosed once a day with corn oil (vehicle control) or the doses mentioned above of DEHP by placing a pipette tip containing the dosing solution into the mouth (**Figure 1**). This method of drug administration was chosen to mimic oral exposure in humans (35). The volume of each dosing mixture in corn oil was adjusted daily based on changes in the dam’s weight.

The DEHP treatment was interrupted after the birth of the offspring rats. After weaning, the rats were separated by sex and kept on standard chow and filtered water until the 90^th^ postnatal day (PND90). Then, the animals were weighed, anesthetized, and euthanized by decapitation. The euthanasia was performed in the morning between 8h00 and 11h00AM. The female offspring were in the metestrus and diestrus phases of the estrous cycle on the day of the euthanasia. Blood, hypothalamus, pituitary, thyroid, and liver were collected, stored at -80°C, and processed as described below.

The experimental protocol was approved by the Ethics Committee on Animal Experimentation of the Hospital Israelita Albert Einstein (CEUA 3326/2018), following the ethical principles in animal research adopted by the National Council for the Control of Animal Experimentation.

### 2.2 Evaluation of gene expression

Hypothalamus, pituitary, thyroid, and liver were homogenized in TRIzol^®^ Reagent (Invitrogen Life Technologies Carlsbad, CA, USA), using Polytron^®^ equipment (KINEMATIC), following the manufacturer’s recommendations (36). In addition, gene expression of the hypothalamus and pituitary (*Tshb, Cga, Trh*), thyroid transcription factors (*Nkx2.1, Pax8, Foxe1*), thyroid differentiation markers (*Slc5a5, Tshr, Tpo, Tg, Mct8*), deiodinases (*Dio1*, *Dio3*), hepatic enzymes (*Ttr, Ugt1a1, Ugt1a6*) was evaluated through RT-qPCR assays. The relative mRNA expression was calculated according to the 2^−ΔΔCt^ method using *Rpl19* mRNA expression as the internal control. Primer sequences are described in **Supplementary Table 1**.

### 2.3 Evaluation of protein content

Hypothalamus, pituitary, and thyroid were homogenized in RIPA lysis buffer (50 mM Tris, pH 7.5; 150 mM NaCl, 1% Nonidet P-40; 0.5% sodium deoxycholate; 1 mM EDTA and 0.1% SDS) supplemented with protease inhibitors (Protease Inhibitor Tablets, Thermo Scientific Pierce™), using Polytron^®^ equipment (KINEMATIC). Western Blotting was performed as previously described (36). Briefly, nitrocellulose membranes were blocked with 3% BSA solution and incubated with specific primary and secondary antibodies, described in **Supplementary Table 2**. Blots were developed using the enhanced chemiluminescence (ECL) kit (Bio-Rad). Densitometric analyses were performed using Image J. 1.4 software (National Institutes of Health). Ponceau staining was used for thyroid total protein normalization, as previously described (37).

### 2.4 Determination of TSH, T3, and T4 serum levels

T_4_ and T_3_ rat serum concentrations were determined by a chemiluminescent immunoassay (Roche). TSH serum levels were determined by a fluorometric immunoassay, as previously described (37).

### 2.5 Statistical analysis

All data are reported as means ± SEM. The number of animals used in the study is indicated in the legends of the figures. Statistical analysis was performed using the GraphPad Prism Software – Version: 6.0. Data was subjected to a normality test (Kolmogorov-Smirnov) and then to unpaired One-Way ANOVA followed by Student-Newman-Keuls post hoc test and/or Dunnet's multiple comparisons test *post hoc* test. Differences were considered statistically significant at P < 0.05.

## 3 Results

### 3.1 Intrauterine exposure to DEHP alters TSH and thyroid hormones serum levels in the F1 female rats

As presented in **Table 1**, the exposure of F1 female rats *in utero* to DEHP did not alter body weight but altered the serum levels of the TSH and thyroid hormones during adulthood. Indeed, the female rats exposed to the highest dose of DEHP treatment during the intrauterine period presented lower serum TSH levels than the control group. Moreover, *in-utero* exposure to DEHP reduced T4 serum levels in the F1 female rats. Interestingly, there was a significant increase in the T3 total serum levels in the animals exposed to the lowest dose of DEHP during the intrauterine period.

**Table 1:** Body weight, thyroid hormones and TSH serum levels in adult F1 female rats that were exposed or not to DEHP during intrauterine period.

	Control	DEHP 0.48 mg/kg/day	DEHP 4.8 mg/kg/day*
**Body weight**	221 ± 4,2	216 ± 6,9	227 ± 4,9
**T_3_ (ng/dL)**	1,20 ± 0,05	1,36 ± 0,02**	1,19 ± 0,02
**T_4_ (μg/dL)**	5,90 ± 0,38	5,01 ± 0,13@	4,14 ± 0,18***
**TSH (ng/mL)**	10,63 ± 1,0	10,72 ± 2,1	5,05± 0,8*

Results are expressed by means ± SEM, n = 5-7 per group. @P = 0.06, * P < 0.05, ** P < 0.01, *** P < 0.001 vs. Control.

### 3.2 Intrauterine exposure to DEHP alters the gene expression and protein content of the F1 female rats’ hypothalamus and pituitary

**Figure 2:**
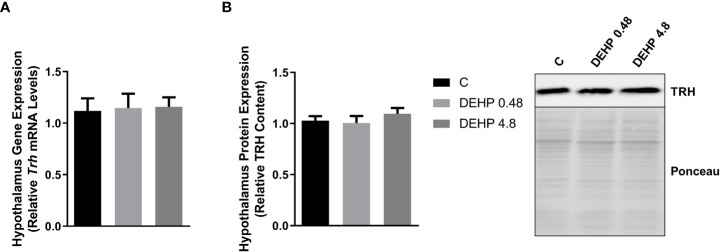
Impact of DEHP exposure during the intrauterine period on TRH expression in the hypothalamus of adult F1 female rats. **(A)** Gene expression (*Trh*) was evaluated by Real-Time PCR and normalized by the expression of the constitutive gene *Rpl19*. **(B)** The protein content of thyrotropin-releasing hormone (TRH) was evaluated by Western Blot and normalized by Western Blot and normalized by Ponceau staining of the membranes. Representative western blots are shown in the right panel. Results are expressed as means ± SEM as fold change or in arbitrary units (AU). Values are expressed as mean ± SEM, in arbitrary units, n = 8-9 animals. P > 0.05 (One-Way ANOVA).

**Figure 3:**
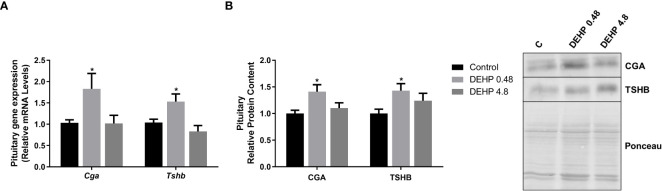
Impact of DEHP exposure during intrauterine period in the expression of alpha and beta subunits of TSH in the pituitary of adult F1 female rats. **(A)** Gene expression (*Cga, Tshb*) was evaluated by Real-Time PCR and normalized by the expression of the constitutive gene *Rpl19*. **(B)** The protein content of CGA and TSHB was assessed by Western Blot and normalized by Ponceau staining of the membranes. Representative western blots are shown in the right panel. Values are expressed as mean ± SEM in arbitrary units, n=10-14 animals. * p < 0.05 vs. Control (One-Way ANOVA).

As demonstrated in **Figure 2**, the intrauterine exposure to DEHP has not altered TRH’s gene and protein expression in the hypothalamus of the F1 female rats during adulthood. Interestingly, exposure to the lowest dose of DEHP significantly increased the gene expression and protein content of the alpha and beta subunits of TSH in the pituitary of the F1 female rats. However, there were no significant alterations in these subunits’ gene/protein content in the animals exposed to the highest dose of DEHP (**Figure 3**).

### 3.3 Intrauterine exposure to DEHP reduces the gene expression and protein content of the F1 female rats’ thyroid gland

**Figure 4:**
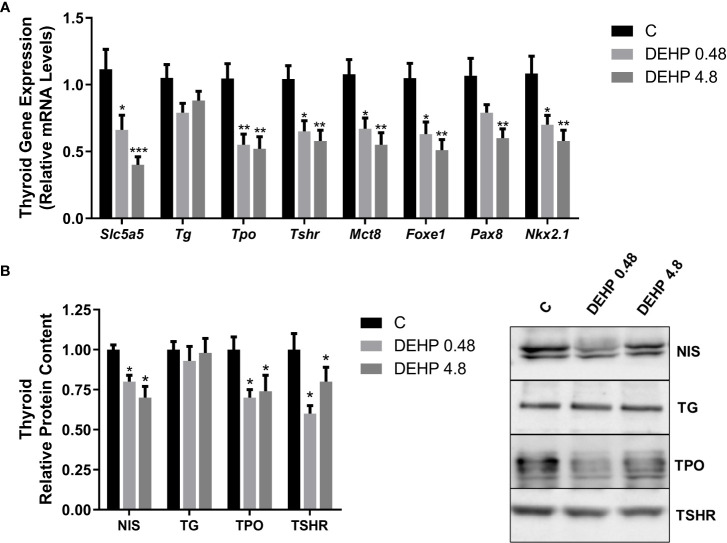
Impact of DEHP exposure during the intrauterine period on thyroid gene and protein expression in adult F1 female rats. **(A)** Gene expression (*Slc5a5, Tg, Tpo, Tshr, Mct8, Foxe1, Pax8, Nkx2.1*) was evaluated by Real-Time PCR and normalized by the expression of the constitutive gene *Rpl19*. **(B)** The protein content of NIS, TG, TPO, and TSHR was evaluated by Western Blot and normalized by Ponceau staining of the membranes (**Supplementary Figure 1**). Representative western blots are shown in the right panel. Values are expressed as mean ± SEM, in arbitrary units, n=8-10. * p < 0.05, ** p < 0.01, *** p < 0.001 vs Control. (One-Way ANOVA).

Intrauterine exposure to both doses of DEHP has also reduced the expression of genes/proteins involved in the synthesis and secretion of thyroid hormones (**Figure 4**). Indeed, there was a significant decrease in the gene expression of *Foxe1* and *Nkx2.1* transcription factors in both treatment doses, while the *Pax8* gene expression was reduced only in the animals exposed to the highest dose of DEHP. In addition, both doses of DEHP exposure reduced the thyroid expression of *Slc5a5, Tpo, Tshr*, and *Mct8* mRNAs compared to the control animals (**Figure 4A**). In agreement, the *in-utero* DEHP-exposed F1 female rats presented a significant reduction in the content of NIS, TPO, and TSHR, critical proteins involved in synthesizing thyroid hormones (**Figure 4B**).

### 3.4 Intrauterine exposure to DEHP alters the gene expression of the F1 female rats’ liver

**Figure 5:**
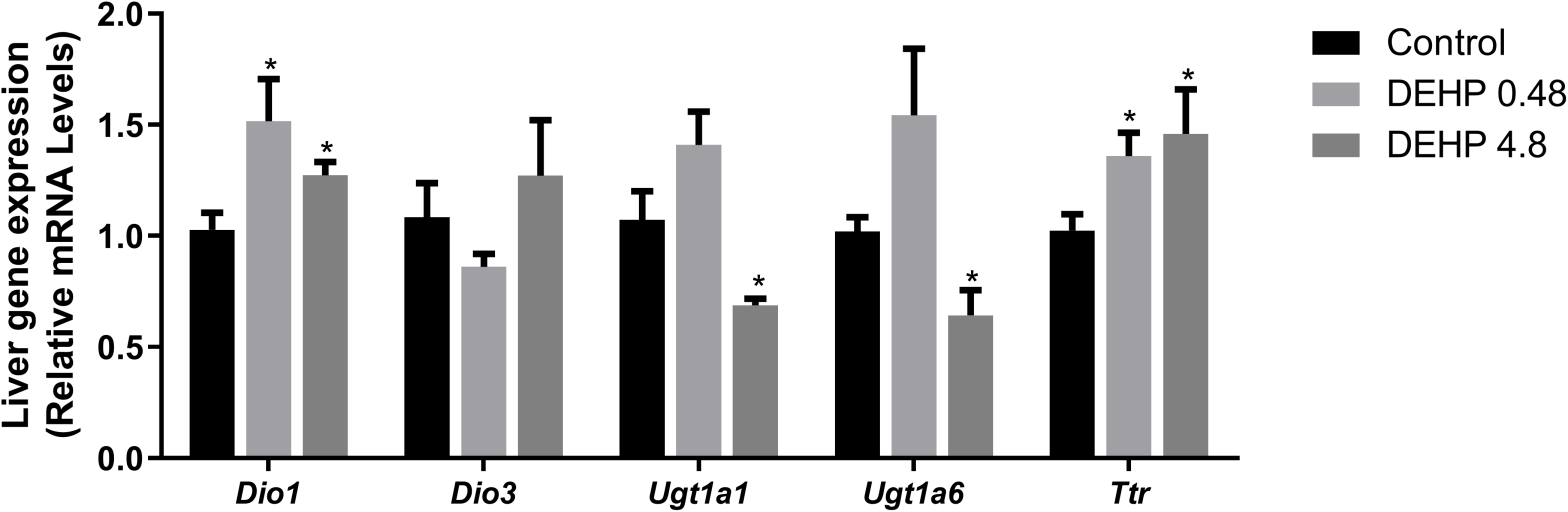
Impact of DEHP exposure during intrauterine period in the liver gene expression in adult F1 female rats. Gene expression (*Dio1, Dio3, Ugt1a, Ugt1a6*, *Ttr*) was evaluated by Real-Time PCR and was normalized by the expression of the constitutive gene *Rpl19*. Values are expressed as mean ± SEM in arbitrary units, n=7-10 animals. * p < 0.05, vs. Control (One-Way ANOVA).

Finally, the gene expression of type I deiodinase (*Dio1*) was significantly increased in the liver of female rats exposed to both doses of DEHP. There was no significant alteration in the expression of type 3 deiodinase (*Dio3*). Moreover, intrauterine exposure to the highest dose of DEHP reduced the mRNA expression of *Ugt1a* and *Ugt1a6*, glucuronosyltransferases involved in the depuration of thyroid hormones. The mRNA expression of the transthyretin (*Ttr*), an essential plasma protein transporter of thyroid hormones, was increased in the liver of DEHP-exposed adult F1 female rats (**Figure 5**).

### 3.5 Intrauterine exposure to DEHP alters TSH serum levels but doesn’t change thyroid hormones serum levels in the F1 male rats

In contrast to the results obtained in the F1 female rats, adult F1 male rats have not presented alterations in the thyroid hormones serum levels. However, both studied doses significantly elevated the TSH serum levels in the DEHP-exposed animals. Notably, the offspring’s body weight did not vary in the DEHP-exposed animals (**Table 2**).

**Table 2:** Body weight, thyroid hormones and TSH serum levels in adult F1 male rats that were exposed or not to DEHP during intrauterine period.

	Control	DEHP 0.48 mg/kg/day	DEHP 4.8 mg/kg/day
**Body Weight**	381 ± 9,4	365 ± 10,1	398 ± 7,7
**T_3_ (ng/dL)**	1,15 ± 0,02	1,18 ± 0,03	1,19 ± 0,02
**T_4_ (μg/dL)**	7,20 ± 0,36	7,20 ± 0,31	6,31 ± 0,44
*TSH (ng/mL)	7,71 ± 0,8	16,08 ± 5,2*	15,14 ± 2,2*

Results are expressed by means ± SEM, n = 5-12 per group. *P < 0.05 vs. Control.

### 3.6 Intrauterine exposure to DEHP alters the gene expression and protein content of the F1 male rats’ hypothalamus and pituitary

**Figure 6:**
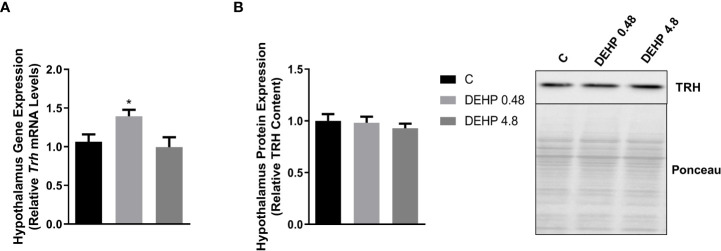
Impact of DEHP exposure during intrauterine period in the TRH expression in the hypothalamus of adult F1 male rats. **(A)** Gene expression (*Trh*) was evaluated by Real-Time PCR and normalized by the expression of the constitutive gene *Rpl19*. **(B)** The protein content of thyrotropin-releasing hormone (TRH) was evaluated by Western Blot and normalized by Ponceau staining of the membranes. Representative western blots are shown in the right panel. Values are expressed as mean ± SEM, in arbitrary units, n = 8-10 animals. * p < 0.05 vs. Control (One-Way ANOVA).

**Figure 7:**
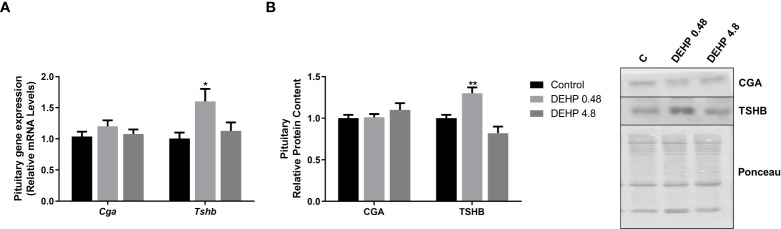
Impact of DEHP exposure during intrauterine period in the expression of alpha and beta subunits of TSH in the pituitary of adult F1 male rats. **(A)** Gene expression (*Cga, Tshb*) was evaluated by Real-Time PCR and normalized by the expression of the constitutive gene *Rpl19*. **(B)** The protein content of CGA and TSHB was evaluated by Western Blot and normalized by Ponceau staining of the membranes.. Representative western blots are shown in the right panel. Values are expressed as mean ± SEM, in arbitrary units, n= 13-14 animals. * p < 0.05, ** p < 0.01 vs. Control (One-Way ANOVA).

Interestingly, the *in-utero* exposure to the lowest dose of DEHP increased the mRNA expression of *Trh* in the hypothalamus of adult F1 male rats, even though there were no significant alterations in the TRH protein content in these animals (**Figure 6**). Furthermore, as demonstrated in **Figure 7**, DEHP exposure has not altered the alpha subunit of TSH expression in the pituitary of the F1 male rats. In contrast, the animals exposed to the lowest dose of DEHP during the intrauterine period presented increased mRNA expression and protein content of the beta subunit of TSH in the pituitary.

### 3.7 Intrauterine exposure to DEHP affects the gene expression and protein content of the F1 male rats’ thyroid gland

**Figure 8:**
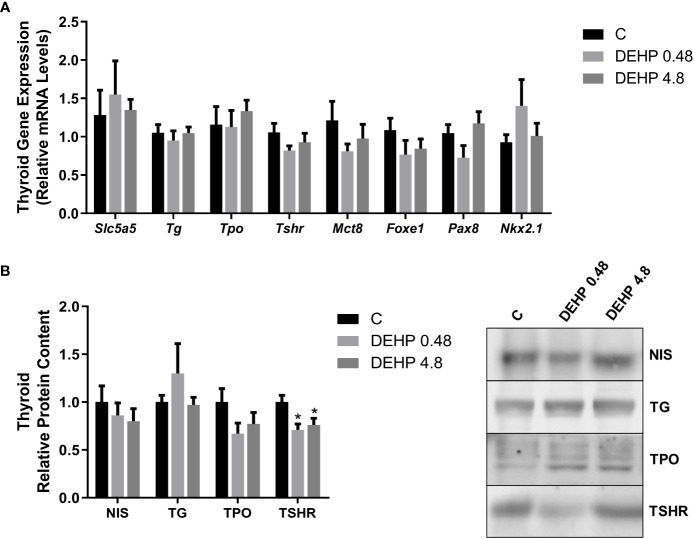
Impact of DEHP exposure during the intrauterine period on thyroid gene and protein expression in the adult F1 male rats. **(A)** Gene expression (*Slc5a5, Tg, Tpo, Tshr, Mct8, Foxe1, Pax8, Nkx2.1*) was evaluated by Real-Time PCR and normalized by the expression of the constitutive gene *Rpl19*. **(B)** The protein content of NIS, NIS, TG, TPO, and TSHR was evaluated by Western Blot and normalized by Ponceau staining of the membranes (**Supplementary Figure 2**). Representative western blots are shown in the right panel. Values are expressed as mean ± SEM, in arbitrary units, n=8-10. * p < 0.05 vs. Control. (One-Way ANOVA).

Interestingly, the exposure of F1 male rats to DEHP *in utero* has not significantly altered the mRNA expression of *Slc5a5, Tg, Tpo, Tshr, Mct8, Foxe1, Pax8*, and *Nkx2.1* (**Figure 8A**). However, DEHP-exposed animals presented a significant decrease in the TSRH protein content in the thyroid (**Figure 8B**).

### 3.8 Intrauterine exposure to DEHP doesn’t alter the gene expression of the F1 male rats’ liver

**Figure 9:**
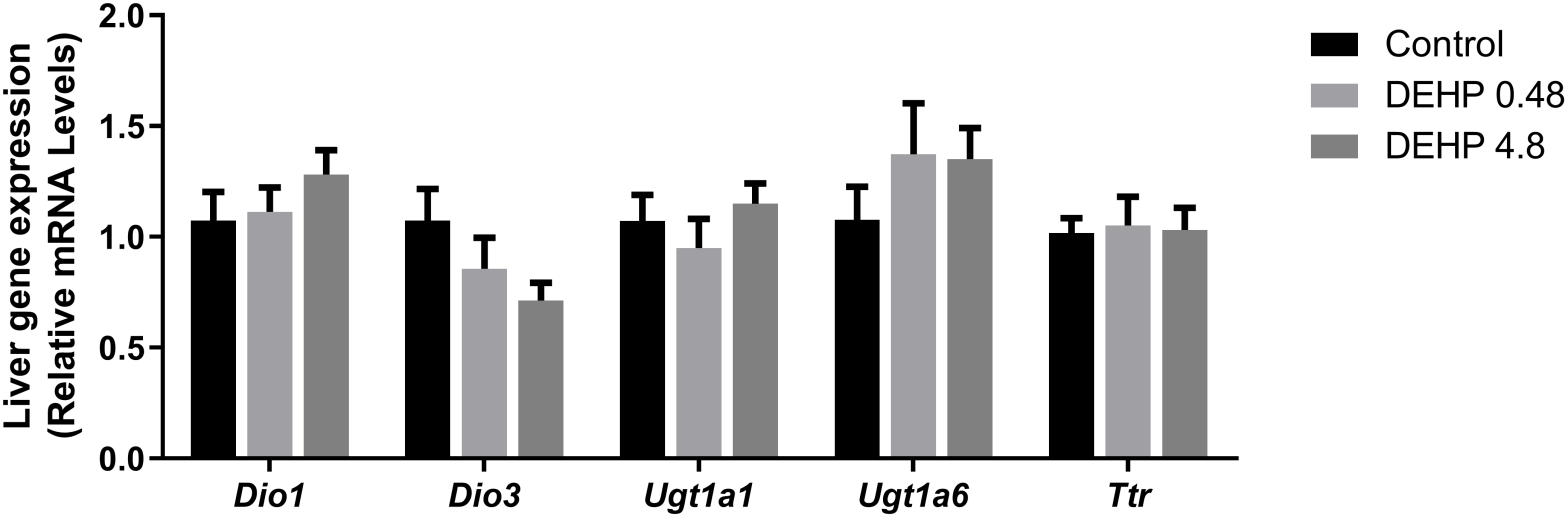
Impact of DEHP exposure during intrauterine period in the liver gene expression in adult F1 male rats. Gene expression (*Dio1, Dio3, Ugt1a, Ugt1a6*, *Ttr*) was evaluated by Real-Time PCR and was normalized by the expression of the constitutive gene *Rpl19*. Values are expressed as mean ± SEM in arbitrary units, n=7-10 animals. p > 0.05 (One-Way ANOVA).

The mRNA expression of *Dio1*, *Dio3, Ugt1a*, *Ugt1a6*, and *Ttr* were not altered in the liver of adult F1 DEHP-exposed male rats compared to the control group (**Figure 9**).

**Figure 10:**
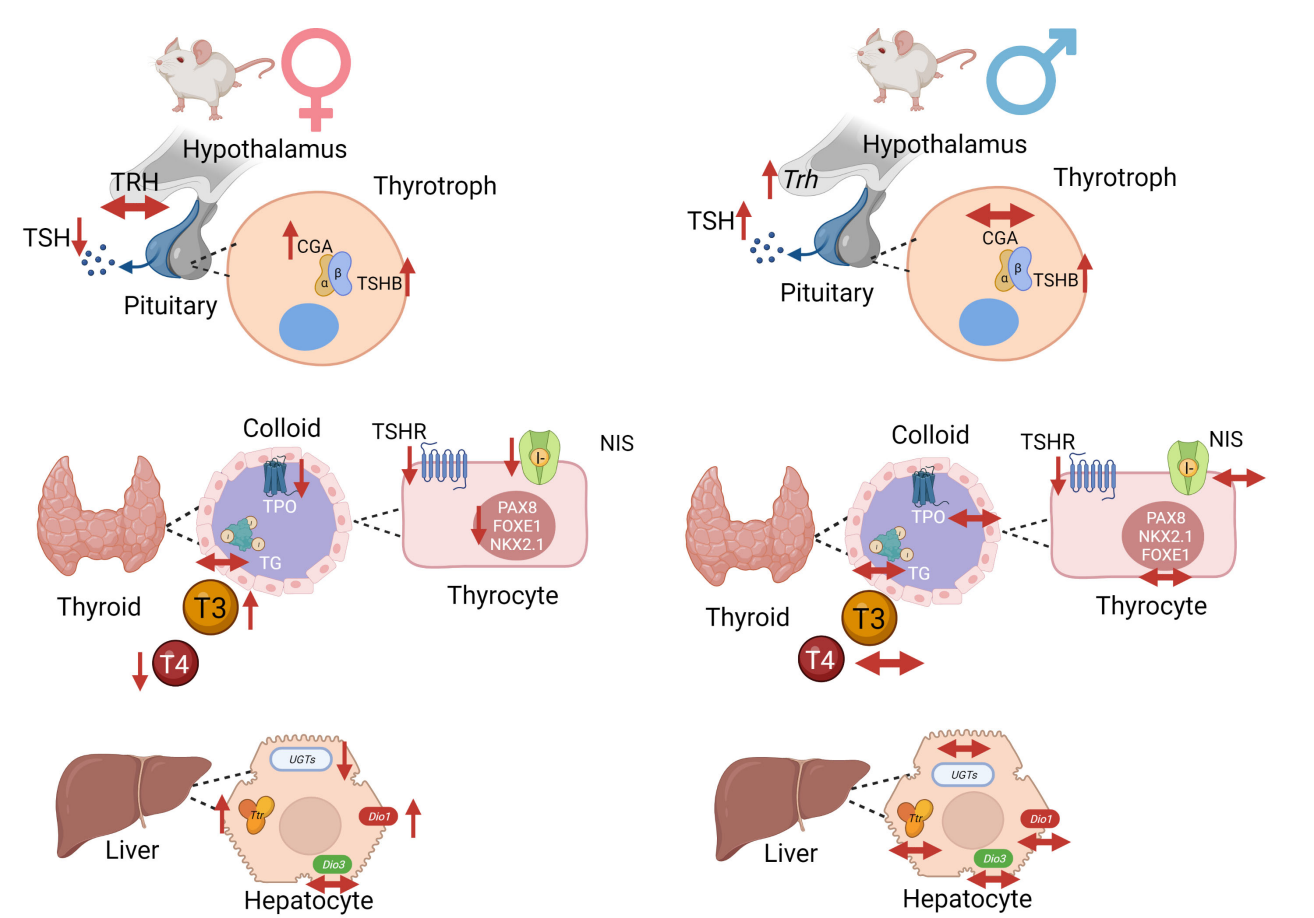
Schematic representation of DEHP effects in the HPT axis of adult F1 female and male offspring animals. In summary, intrauterine exposure to DEHP induced hypothyroidism in adult female offspring rats and subclinical hypothyroidism in adult male offspring rats. Created with BioRender.com.

In summary, **Figure 10** presents a schematic representation of the deleterious effects of intrauterine exposure to DEHP in the HPT axis function of F1 offspring during adulthood.

## 4 Discussion

The data presented in this study demonstrated that intrauterine exposure to DEHP disrupts the HPT axis of the offspring rats during adulthood. Our results also suggest that the male and female offspring present different sensitivity to DEHP exposure.

The disruptive effects of DEHP exposure in the HPT axis were previously reported in the literature (38–40). However, although these studies were very elucidative about the deleterious effects of DEHP on thyroid function, the studied doses were much higher than those used herein. Moreover, most of the previous studies were performed in male rats. It is well known that the prevalence of thyroid diseases is higher in women than in men (41). For that reason, we aimed to evaluate the consequences of DEHP exposure in both genders. Therefore, our study was the first to describe the effects of intrauterine exposure to DEHP in the programming of the HPT axis both in male and female offspring.

The adverse effects triggered by intrauterine exposure to DEHP were previously reported in the reproductive function and neurobehavioral of the offspring (32, 42). However, the impact of this exposure directly on thyroid function is poorly described (33, 43). Therefore, this is a limitation of the present study since it is difficult to compare the obtained results with those presented in the literature.

In the hypothalamus of the adult F1 female rats, there was no alteration in the gene/protein expression of TRH. Contrary to these results, the male offspring rats presented increased *Trh* mRNA expression in the lowest treatment dose without significant alterations in the TRH protein content. Sun et al. also observed increased TRH content in the hypothalamus of male rats directly exposed to high doses of DEHP (50 to 500 mg/kg/day) (39).

It is well described that thyroid hormones exert a negative feedback loop in the hypothalamus and pituitary (44). Therefore, our data suggest an impairment in the HPT axis function both in the male and female offspring since there was an increased expression of *Trh* in the hypothalamus of the male rats even in the absence of thyroid hormones alterations, and the *Trh* expression was not upregulated in the females with reduced T4 levels.

In the pituitary, the adult F1 female rats presented increased gene/protein expression of CGA, especially in the lowest dose of DEHP treatment. It is well known that the glycoprotein hormones, such as TSH, FSH, and LH, are composed of a common alpha-subunit and a specific beta-subunit, which confers biological specificity to these hormones. (45). Therefore, the increased expression of CGA in the female offspring rats’ pituitary suggests that the synthesis and secretion of FSH and LH are potentially altered in the DEHP-exposed animals. These results follow previous data about the disruptive effects of DEHP exposure, including during the intrauterine period, in the hypothalamus-pituitary-ovary axis function and the reproductive outcomes in female rats (35, 42). Interestingly, the exposure to DEHP during the intrauterine period decreased TSH levels only in the F1 female offspring exposed to the highest dose of treatment

The exposure to DEPH increased the content of the TSHB in the adult F1 male rats. This result was consistent with the increased TSH serum levels that were observed in the DEHP-exposed animals. Even though the effects of DEHP exposure on the expression/secretion of TSH are still controversial, Dong et al. demonstrated that the perinatal exposure to DEHP (30 to 750 mg/kg/day) increased the TSH serum levels of the pups at PND 7, PND 14, and PND 21 (43). The results also agree with previous results that demonstrated increased TSH levels in the male offspring rats of rat dams exposed to 600 mg/kg/day of DEHP (33). Moreover, our results are consonant with previous studies carried out in zebrafish that reported a significant increase in the *Tshb* expression MEHP-exposed animals (40 and 200 μg/L) (46). Nevertheless, studies performed with male rats chronically exposed to DEHP (600 mg/kg/day) presented decreased levels of TSH. (40). These discordant results indicate that the exposure period is crucial to determine the DEHP-triggered effects on the TSH expression in the pituitary.

TSH is the primary regulator of thyroid morphology and all the steps involved in thyroid hormone synthesis. TSH triggers its effects through the binding in the TSHR expressed in the basolateral membrane of thyrocytes (47). As described herein, the intrauterine exposure to DEHP triggered different results in the thyroid of the adult F1 female and male offspring rats. In the F1 female rats, there was a significant reduction in the expression of genes/proteins involved in the synthesis and secretion of thyroid hormones in both studied doses of DEHP, which was consistent with the reduced serum levels of T4 in these animals. However, in the males, thyroid gene expression was not altered by DEHP exposure, although TSHR expression was reduced in these animals. Moreover, the thyroid hormone levels were not changed in the adult F1 males, although TSH levels were significantly increased in both studied doses.

The disruptive effect of DEHP on TSH, TSH signaling pathway, and thyroid hormone levels have been previously reported, especially in male rats. Indeed, it has been demonstrated that the exposure of rats to high doses of DEHP (50 and 500 mg/kg/day) for two weeks reduced T3 and T4 levels and the *Tshr* mRNA expression in the thyroid gland (48). Rats chronically and directly exposed to DEHP (250, 500, or 750 mg/kg/day) presented reduced levels of thyroid hormones or increased levels of TSH (26, 27, 49).

Indeed, thyroid transcription factors are regulated by TSH and are critical to maintaining thyroid function and controlling the expression of thyroid differentiation genes (50, 51). Thus, the significant reduction of *Pax8*, *Nkx2.1*, and *Foxe1* mRNA in the thyroid of F1 female rats could justify the decrease in the expression of the other thyroid genes. Additionally, the reduced TSH serum levels observed in the females exposed to the highest dose of DEHP could also contribute to the reduced thyroid transcriptional activity. Therefore, in the males, the absence of thyroid gene expression regulation, even with high serum circulating levels of TSH, suggests that the thyroid gland is hypofunctional in these animals. This hypothesis is strengthened by the reduced expression of TSHR in the thyroid of DEHP-exposed animals.

The impact of DEHP exposure on thyroid gene expression and function is still inconclusive. In fact, previous studies demonstrated that chronic exposure to high doses of DEHP (150, 300, 600 mg/kg/day) induced a stimulatory effect on *Slc5a5, Tpo, Tshr*, and *Tg* mRNA expression in rats (40). In agreement, rats perinatally exposed to DEHP also presented increased expression of genes/proteins involved in the biosynthesis of thyroid hormones (43). On the other hand, Kim et al. demonstrated reduced expression of TSHR in male rats exposed to DEHP (50 and 500mg/kg/day) for 14 days (48). These results are in accordance with previous studies that demonstrated reduced expression of NIS and TPO in rats chronically treated with high doses of DEHP (500 and 750 mg/kg/day) (27). Although this is the first study to evaluate the impact of *in utero* DEHP exposure in the thyroid gene/protein expression of the offspring during adulthood, previous studies have clearly demonstrated impairment in the thyroid hormones secretion and action in DEHP-exposed animals during critical windows of susceptibility (32, 33, 52). These data reinforce that gender, the treatment dose, and the period of exposure are crucial to determining the effects of DEHP on the HPT axis.

Although the female rats presented decreased expression of genes/proteins involved in thyroid hormone production, T3 levels were increased in the lowest treatment dose. Previous data indicated increased T3 serum levels and decreased T4 levels in animals exposed to DEHP (300 mg of DEHP/kg/day) (40, 53).

Thyroid hormone serum levels depend on the thyroid secretion rate and the expression/activity of deiodinases in the peripheral tissues (54). Therefore, the increased expression of *Dio1* mRNA in the liver of F1 female rats could contribute to the increased circulating levels of T3. Conversely, there were no significant alterations in the *Dio1* mRNA expression in the liver of adult F1 male rats, suggesting a sexually different response in the liver of DEHP-exposed animals.

The regulation of deiodinases expression by DEHP has already been reported in the literature. Indeed, the exposure of male rats to DEHP (600 mg/kg/day) for six months increased the *Dio1* mRNA expression in the liver (55). Perinatal exposure to DEHP was also related to increased *Dio1* mRNA levels in PND14 and PND21 rats (43). Furthermore, zebrafish exposed to significant environmental concentrations of DEHP presented increased expression of *Dio1* and *Dio 2* transcripts (46).

Transthyretin (*Ttr*) is the main transport protein of thyroid hormones in rats (56). In the present study, *in-utero* exposure to DEHP increased the *Ttr* mRNA expression in the liver of female offspring but not in the male one. In contrast, previous studies have demonstrated a suppressive effect of chronic DEHP exposure in the TTR expression, which was associated with decreased thyroid hormone serum levels (27). Future studies are needed to unravel the sexually dimorphic response of *Ttr* mRNA expression in DEHP-exposed animals and elucidate the molecular mechanisms involved in the differential regulation of *Ttr* mRNA expression in animals exposed during critical periods of development or during adulthood.

Finally, UDP-glucuronyltransferases (UGTs) enzymes catalyze the glucuronidation and peripheral degradation of thyroid hormones (56). The regulation of the expression of these enzymes presented a different response in the DEHP-exposed male and female offspring. Future studies might elucidate the molecular pathways in regulating these crucial enzymes in DEHP-exposed animals.

This study has some limitations. First, although we reported several alterations in the gene/protein expression in the HPT axis glands, we couldn’t wholly elucidate the molecular mechanisms involved in this disruption. We have preliminary data suggesting the participation of epigenetic mechanisms in regulating thyroid transcriptional activity, but additional studies will be needed to elucidate their involvement in the disruption of thyroid gene transcription. Moreover, the morphological analysis of the thyroid gland would contribute to understanding if the disruption of the thyroid function in the offspring animals is related to the impairment of the follicle’s structure.

In summary, the data presented herein indicated, for the first time, that intrauterine exposure to DEHP causes long-term damage in the HPT axis function both in the male and female offspring. Furthermore, the results suggest that exposure to DEHP programs the offspring’s thyroid function, increasing their susceptibility to developing thyroid dysfunctions during adulthood.

## Data availability statement

The raw data supporting the conclusions of this article will be made available by the authors, without undue reservation.

## Ethics statement

The animal study was reviewed and approved by Ethics Committee on Animal Experimentation of the Hospital Israelita Albert Einstein (CEUA 3326/2018).

## Author contributions

ES-V performed the treatments and experiments, processed the tissues, collected the data, performed the analysis, and wrote the paper. GH processed the tissues, collected the data, and performed the analysis. RS collected the data, performed the analysis. CS-N conceived and designed the analysis, performed the treatments, and wrote the paper. All authors contributed to the article and approved the submitted version.
