# Supplementary material for: Intrauterine exposure to di(2-ethylhexyl) phthalate (DEHP) disrupts the function of the hypothalamus-pituitary-thyroid axis of the F1 rats during adult life
Source: Front Endocrinol (Lausanne). 2023 Jan 13;13:995491. doi: 10.3389/fendo.2022.995491 (PMC9880230; doi:10.3389/fendo.2022.995491)
Supplement: Supplementary file 1 [file DataSheet_1.docx]

**SUPPLEMENTAL DATA. *"*** ***Intrauterine exposure to di(2-ethylhexyl) phthalate (DEHP) disrupts the function of the hypothalamus-pituitary-thyroid axis of the F1 rats during adult life."***

**Supplemental Table 1 –** Primer sequences.

| mRNA | Forward | Reverse |
| --- | --- | --- |
| **Slc5a5** | AGCCTCGCTCAGAACCATTC | GTGTACCGGCTCCGAGGAT |
| **Pax8** | CTGTCTCAGGCCAAGTC | CAGCCTGCTGAGTTCTCCAT |
| **Nkx2.1** | ACCTTACCAGGACACCATGC | TTCTTGCTCACGTCCCCCAG |
| **Foxe1** | CTGGGACCCTGCTACAATCC | TCACATGGCAGACACGAACC |
| **Tshr** | GGCTGCTGGCTGCTTCTTTT | TCAGACGCATGATCAAAATGAAA |
| **Tg** | CTCAGGACGATGGGCTTATCA | GTTCGGCCTTGGCTTTCTTC |
| **Tpo** | ACAGTTCTCCACGGATGCACTA | GGCAAGCATCCTGACAGGTT |
| **Dio1** | ATTTGACCAGTTCAAGAGACTCG | GGCGTGAGCTTCTTCAATGTA |
| **Mct8** | AGCCTGCGCTACTTCACCTA | GGCCAGCTTGATTCTGTCTC |
| **Tshb** | GGCAAACTGTTTCTTCCCAA | GTTGGTTTTGACAGCCTCGT |
| **Cga** | CACTCTGGCATTTCCCATTA | GCCAGGTCCAAGAAGACAAT |
| **Trh** | CGGTGCTGCCTTAGACTCCTGGA | GCCGGGGTGCTGTCGTTTGT |
| **Rpl19** | CCAATGAAACCAACGAAATCG | TCAGGCCATCTTTGATCAGCTT |

**Supplementary Table 2**. Primary Antibodies List.

| Protein | Source | Specie | Dilution |
| --- | --- | --- | --- |
| TRH | Abcam | Rabbit | 1:1000 |
| CGA | NIH | Rabbit | 1:3000 |
| TSHΒ | NIH | Rabbit | 1:3000 |
| TSHR | Abcam | Rabbit | 1:1000 |
| NIS | Donated by Dr. Nancy Carrasco | Rabbit | 1:3000 |
| TG | Abcam | Mouse | 1:2000 |
| TPO | Santa Cruz | Mouse | 1:500 |

Abbreviations: TRH: thyrotropin-releasing hormone; CGA: alpha subunit of thyroid stimulating hormone; TSHB: beta subunit of thyroid stimulating hormone; TSHR: thyroid stimulating hormone receptor; NIS: sodium-iodine symporter; TG: thyroglobulin; TPO: thyroid peroxidase.

**Supplementary Figure 1**

D.

C.

B.

A.

**Supplementary Figure 1 –** Ponceau S staining was used as loading control of the immunoblots from nitrocellulose membranes of NIS (A), TG (B), TPO (C), and TSHR (D) in the thyroid of Control and DEHP-exposed animals (0.48 and 4.8 mg/kg/day, respectively).

**Supplementary Figure 2**

D.

C.

B.

A.

**Supplementary Figure 2 –** Ponceau S staining was used as loading control of the immunoblots from nitrocellulose membranes of NIS (A), TG (B), TPO (C), and TSHR (D) in the thyroid of Control, DEHP-exposed animals (0.48 and 4.8 mg/kg/day, respectively).
